# A Monstrosity

**Published:** 1889-09

**Authors:** C. C. Lyon

**Affiliations:** Carmel, Ark.


### A MONSTROSITY.

CARMEL, ARK., August 17, '89.

*Editors Atlanta Medical and Surgical Journal, Atlanta, Ga.:*

GENTLEMEN:—Some time ago I was called to see an infant female, aged two weeks, of German parentage, the father of the child informing me that it had a tumor on its back. Upon examination I found an extra *pelvis* at the upper portion of the lumbar region to the left of the vertebræ. The labia were perfect but the *clitoris* was simply a membranous formation filled with bright bloody serum, filling the cavity between the labia or rather distending the labia. I intended operating as soon as practicable but the child died in about two weeks, during the hard, stormy weather then in vogue. Fraternally, C. C. LYON.

